# Novel enzyme-linked aptamer-antibody sandwich assay and hybrid lateral flow strip for SARS-CoV-2 detection

**DOI:** 10.1186/s12951-023-02191-9

**Published:** 2024-01-03

**Authors:** Shih-Wei Wu, Ying-Ju Chen, Yu-Wen Chang, Cheng-Yang Huang, Biing-Hui Liu, Feng-Yih Yu

**Affiliations:** 1 Graduate Institute of Toxicology, College of Medicine, National Taiwan University, No.1, Sec. 1, Jen Ai Rd, Taipei, 100 Taiwan; 2 School of Medicine, Chung Shan Medical University, No.110, Sec. 1, Chien Kuo N. Rd, Taichung, 402 Taiwan; 3 Department of Biomedical Sciences, Chung Shan Medical University, No.110, Sec. 1, Chien Kuo N. Rd, Taichung, 402 Taiwan; 4 Department of Medical Research, Chung Shan Medical University Hospital, No.110, Sec. 1, Chien Kuo N. Rd, Taichung, 402 Taiwan

**Keywords:** SARS-CoV-2, Oligonucleotide aptamer, Monoclonal antibody, Hybrid enzyme-linked aptamer-antibody sandwich assay, Hybrid lateral flow strip

## Abstract

We have successfully generated oligonucleotide aptamers (Apts) and monoclonal antibodies (mAbs) targeting the recombinant nucleocapsid (N) protein of SARS-CoV-2. Apts were obtained through seven rounds of systematic evolution of ligands by exponential enrichment (SELEX), while mAbs were derived from the 6F6E11 hybridoma cell line. Leveraging these Apts and mAbs, we have successfully devised two innovative and remarkably sensitive detection techniques for the rapid identification of SARS-CoV-2 N protein in nasopharyngeal samples: the enzyme-linked aptamer-antibody sandwich assay (ELAAA) and the hybrid lateral flow strip (hybrid-LFS). ELAAA exhibited an impressive detection limit of 0.1 ng/mL, while hybrid-LFS offered a detection range of 0.1 – 0.5 ng/mL. In the evaluation using ten nasopharyngeal samples spiked with known N protein concentrations, ELAAA demonstrated an average recovery rate of 92%. Additionally, during the assessment of five nasopharyngeal samples from infected individuals and ten samples from healthy volunteers, hybrid-LFS displayed excellent sensitivity and specificity. Our study introduces a novel and efficient on-site approach for SARS-CoV-2 detection in nasopharyngeal samples. The reliable hybrid Apt-mAb strategy not only advances virus diagnostic methods but also holds promise in combating the spread of related diseases.

**Supplementary Information:**

The online version contains supplementary material available at 10.1186/s12951-023-02191-9.

## Introduction

Emerging infectious diseases such as SARS (severe acute respiratory syndrome coronavirus) [1], MERS (Middle East respiratory syndrome coronavirus) [2] and SARS-CoV-2 (COVID-19) [3] have swiftly surfaced, presenting a substantial global public health hazard. The accelerated transmission of infectious agents across borders and continents can be attributed to the heightened levels of global travel and trade, resulting in disease outbreaks and epidemics in previously unaffected regions and populations. Consequently, the development of assays to impede disease transmission becomes imperative in effectively managing infectious diseases and safeguarding public health. Furthermore, the availability of precise, prompt, and highly sensitive assays facilitates early detection and identification of infectious agents, playing a vital role in curbing the spread of diseases.

Presently, diagnostic tools for major infectious diseases can be broadly classified into three categories: molecular diagnostics for viral RNA, serology tests for anti-viral antigens immunoglobulins, and rapid point-of-care tests for viral antigens [4]. Among these, the real time reverse transcriptase-polymerase chain reaction (RT-PCR) is the widely accepted gold standard procedure utilized in clinical testing. However, despite its commendable sensitivity and specificity, false-negative results are not uncommon, and the accuracy of the test can also be influenced by the site and quality of clinical sampling. Additionally, the requirement for specialized equipment and trained personnel makes real time RT-PCR an expensive and time-consuming process, posing a significant challenge for low and middle-income countries. To address these challenges, there is a pressing need to develop rapid and user-friendly immunoassays for the detection of these infectious diseases [5]. Enzyme-linked immunosorbent assay (ELISA), lateral flow immunoassay (LFIA), and chemiluminescent immunoassay (CLIA) are the three most commonly used immunoassay methods for the detection of infectious diseases [6]. Among them, rapid antigen detection tests are widely employed due to their low cost and high time-effectiveness for detection. However, despite their advantages, several studies have indicated that the sensitivity of commercial rapid viral antigen detection tests is not high enough to be used alone for diagnosis [7]. Therefore, there is an urgent need to develop a rapid antigen detection test with higher sensitivity that can be used independently for diagnosis, thereby significantly enhancing the management of infectious diseases.

Antibodies play a vital role in immunoassays, but they possess certain drawbacks. These include the need to derive them from experimental animals, the requirement for aseptic procedures, the necessity for skilled professionals to generate them, and their limited tolerance for antigenic configurations. These factors contribute to a lengthy and costly production process. Consequently, oligonucleotide aptamers (Apts) have emerged as a promising alternative for developing aptamer assays. Aptamers can adopt a unique tertiary structure through folding, enabling them to bind specific targets with a high affinity [8–10]. Aptamers are often referred to as chemical antibodies [10]. In comparison to antibodies, aptamers offer numerous advantages. They can be screened in vitro, conjugated with multiple molecules, are non-immunogenic, easily synthesized chemically, and exhibit minimal batch variations. Additionally, aptamers are biochemically stable and can be amplified and produced by PCR. The systematic evolution of ligands by exponential enrichment (SELEX) is a common method for screening aptamers, requiring only simple and rapid chemical synthesis. Typically, nucleic acids with high affinity to the target can be obtained after 10–15 rounds of screening, and then nucleic acid sequencing is performed to verify the sequence of the aptamer. Aptamer-based assays have garnered attention as a potential diagnostic tool [11–13].

Antibodies and aptamers possess distinct characteristics that complement each other in various analytical applications. To leverage their complementary properties and overcome the limitations associated with each biomolecule, hybrid aptamer-antibody analysis methods have been developed. This innovative approach enhances the sensitivity and specificity of analysis while enabling the simultaneous detection of multiple targets [11, 13]. In this approach, antibodies or aptamers are immobilized on a solid support, and the sample containing the target molecules is introduced. The antibodies or aptamers selectively capture the target molecules, which are subsequently detected using labeled antibodies or aptamers that specifically recognize the captured targets.

**Fig. 1:**
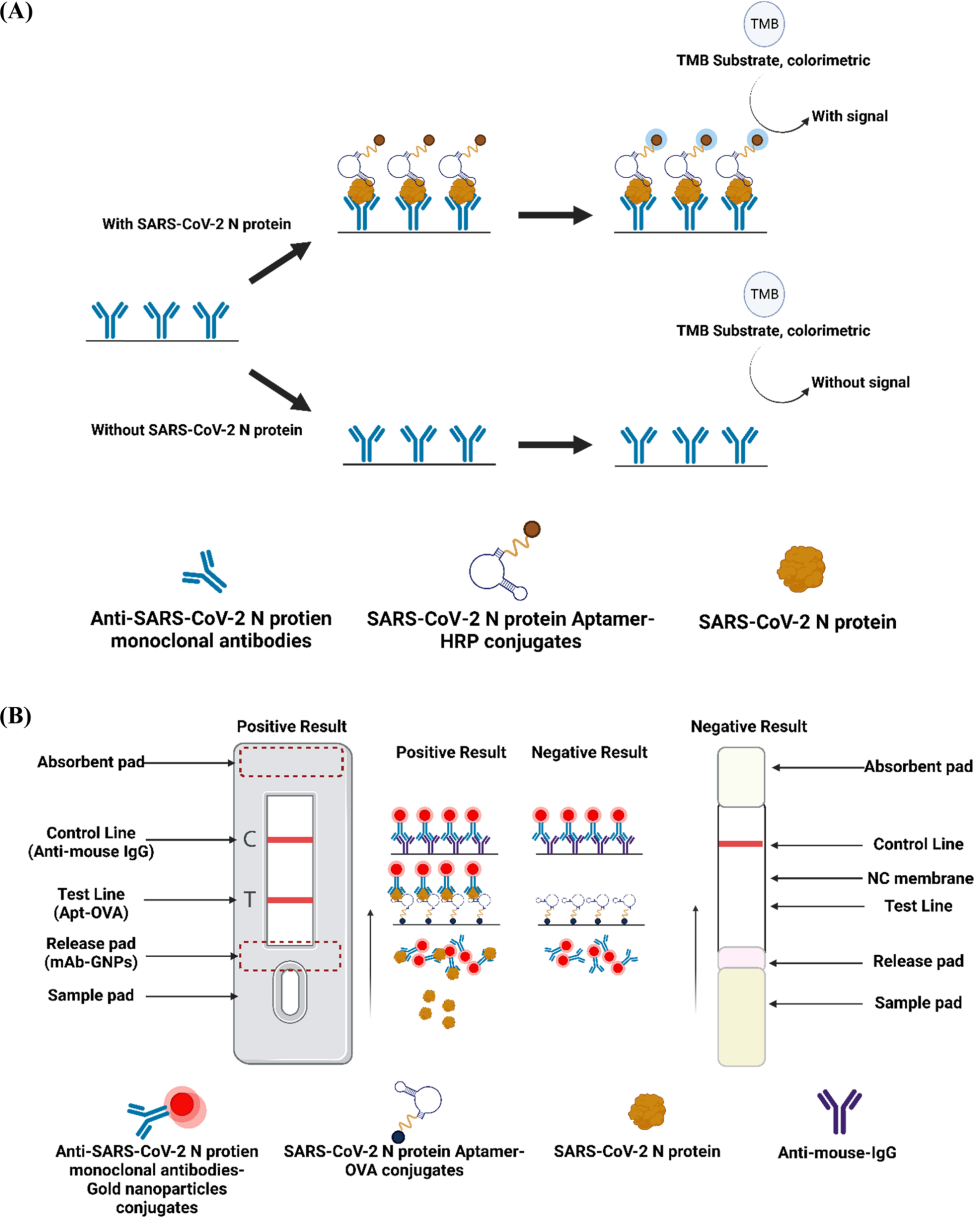
Schematic diagrams of the **A** hybrid ELAAA and **B** the hybrid-LFS

Hence, this study aims to use SARS-CoV-2 as a model to develop a novel, highly sensitive, enzyme-linked aptamer-antibody sandwich assay (ELAAA) and a hybrid lateral flow strip (hybrid-LFS) for detecting the N protein in nasopharyngeal samples (Fig. 1). While previous studies have successfully developed SARS-CoV-2 antibody-based analysis methods for N protein detection and aptamer-based analysis methods for spike protein (S) detection in nasopharyngeal samples or antibody detection in serum samples [14, 15], none of these methods have demonstrated sufficient sensitivity for effective prevention and control. This research introduces a more convenient and cost-effective aptamer-antibody hybrid method for aiding in the prevention and control of SARS-CoV-2. Moreover, this aptamer-antibody hybrid analysis method holds promise as a robust detection tool for various infectious diseases, enabling regulators to mitigate the rapid spread of infectious diseases effectively.

## Materials and methods

### Materials

Nucleocapsid protein standard was purchased from LEADGENE Biomedical Inc. (Tainan, Taiwan). An Easypack Developer's kit was purchased from MDI membrane technologies (Ambala, India). The double axes programmable controller (HGS510), used for the lineation of the test and control lines of the membrane, was purchased from AUTOKUN (Hangzhou, China). The lateral flow strip scan reader and strip cassette were obtained from Taiwan Advance Bio-Pharmaceutical Inc. (Taipei, Taiwan). The capillary electrophoresis (Qsep1-Lite) was obtained from Bioptic Inc. (Taipei, Taiwan). All other reagents and materials used in the experiment are listed in supplementary section (Materials). All organic solvents and chemicals used were of reagent grade or better. The DNA oligonucleotide library, SELEX forward and reverse primers were synthesized and purified by Mission Biotech (Taipei, Taiwan).

### Expression of SARS-CoV-2 N protein

The SARS-CoV-2 N protein expression vector, obtained from the laboratory of Brian J. Geiss, Ph.D and purchased from Kerafast (Boston, USA), was transformed into *Escherichia coli* BL21 cells. The cells were cultured in LB medium at 37 °C until the optical density reached 0.6 at 595 nm. Subsequently, isopropyl-β-thiogalactopyranoside (IPTG) solution (0.5 mM) was introduced into the LB medium to initiate protein expression. Following overnight induction at 25 °C, the cells were harvested by centrifugation at 12,000 rpm for 30 min, and the resulting pellet was dissolved in a 50 mM Tris–HCl buffer (pH 8.0) containing 150 mM NaCl and 1 mM PMSF. The cell solution was then lysed through sonication and subjected to centrifugation at 13,000 rpm for 30 min. The resulting pellet was dissolved in a 50 mM Tris–HCl buffer (pH 8.0) containing 150 mM NaCl and 8 M urea. The mixture underwent additional sonication to dissolve the expression protein present in the inclusion body, and a 100% ammonium sulfate solution (pH 9.0, final concentration of 60%) was added to facilitate the refolding of the N protein. After a 30-min precipitation at room temperature, the reaction mixture was centrifuged at 13,000 rpm for 30 min, and the resulting pellet was dissolved in deionized water. The refolding reaction mixture was subsequently dialyzed against a 50 mM Tris–HCl buffer (pH 8.0) containing 150 mM NaCl and 10 mM imidazole. The N protein was purified using Ni–NTA His-Tag purification resin (Thermo Fisher Scientific Inc, USA), and the eluted fractions were collected and further dialyzed against 0.01 M PBS. The concentration of the N protein was determined by measuring the absorbance at 280 nm using a Bio-Rad protein assay system with bovine serum albumin (BSA) serving as a standard.

### Production of monoclonal antibodies against SARS-CoV-2

#### Generation of monoclonal antibodies

Monoclonal antibodies (mAb) against SARS-CoV-2 N protein were generated following a previously reported protocol [16]. Female BALB/c mice, aged four weeks, were intraperitoneally injected with 25 µg of SARS-CoV-2 N protein in 250 µL of 0.01 M PBS emulsified with an equal volume of Freund’s complete adjuvant. Subsequently, the mice were given weekly boosters with 25 µg of SARS-CoV-2 protein in 250 µL of 0.01 M PBS without adjuvant, and serum was collected from the tail vein. The sensitivity and specificity of the antibodies were evaluated using an indirect ELISA (iELISA), as outlined in supplementary section (Indirect ELISA procedure). Following an eight-week period, the mouse exhibiting the highest antibody sensitivity and specificity was chosen, and its spleen was aseptically removed. Subsequently, the spleen cells were fused with 2 × 10^7^ myeloma cells (NS-1) utilizing PEG-1500, and the resultant cells were distributed into 96-well tissue culture plates. Hybridoma cells producing specific antibodies against the SARS-CoV-2 N protein were identified using the iELISA and isolated via the limiting dilution method.

#### Purification of monoclonal antibodies

Following the limiting dilution step, the hybridoma cells that secreted specific antibodies against the N protein were selected. The monoclonal antibodies were then precipitated twice with 50% ammonium sulfate solution, dialyzed in 50 mM Tris–HCl buffer (pH 8.0) containing 100 mM NaCl, and further purified using a protein A column. The mAb solution was added to the column, and after an overnight reaction at 4 °C, unbound antibodies were washed off with 50 mM Tris–HCl buffer (pH 8.0) containing 100 mM NaCl. The antibodies bound to protein A were eluted using 100 mM glycine solution (pH 2.7), and the eluted solutions were dialyzed against 0.01 M PBS.

#### SDS-PAGE and western blotting

The purified expression N protein was resolved on a 10% gel at 120 V for 1 h before being transferred to PVDF membranes for 1.5 h at 100 V. The membranes were then blocked with 0.5% BSA in PBST (0.05% Tween 20 in 0.01 M PBS) at room temperature for 30 min with shaking. Next, the membranes were incubated with the anti-N protein from mouse serum or monoclonal antibodies in BSA-PBST (0.1% BSA and 0.05% Tween in PBS) at room temperature for 1 h with continuous shaking. After washing three times with PBST for 5 min each, the membranes were incubated with goat anti-mouse-HRP conjugates diluted in PBST at room temperature for 1 h with steady shaking. Finally, the membranes were washed three times with PBST and visualized using the Immobilon ECL western HRP substrate (Merck, Germany).

### Generation of the SARS-CoV-2 aptamer

#### Preparation of SARS-CoV-2 N protein affinity column

To generate the N protein aptamer, an N protein affinity column was prepared using CarboxyLink^TM^ Coupling gel (Thermo Fisher Scientific Inc, USA) in accordance with the product manual. In brief, a 2 mL portion of coupling gel was introduced into the column, washed with 10 mL of 0.1 M MES buffer (pH 4.7) containing 0.9% NaCl. Then, 1 mL of N protein solution (1 mg dissolved in 1 mL of 0.1 MES buffer) and 1-Ethyl-3-[3-dimethylami-nopropyl]-carbodiimide hydrochloride solution (EDC, 1 mg dissolved in 0.5 mL of 0.1 MES buffer) were added. The reaction was allowed to proceed overnight at 4 °C. After passing the reaction mixture through the column, any unbound N protein was removed by washing with 20 mL of 1 M NaCl solution. Finally, the N protein affinity column was stored in 20% ethanol until it was ready for use.

#### Generation of SARS-CoV-2 N protein aptamers using SELEX method

To generate N protein aptamers, the procedure of systematic evolution of ligands by exponential enrichment (SELEX) method was followed the reported previously with slight modifications [17, 18]. The single-stranded DNA (ssDNA) oligonucleotides contained a central randomized region of 40 nucleotides flanked by two primer regions. The forward primer (5’-ATAGGAGTCACGACGACC-3’) and the reverse primer (5’-GTCAAGAGGTAGACGCAC-3’) were used for PCR [19]. All oligonucleotides were synthesized by Mission Biotech Inc. (Taipei, Taiwan). In the first round, the synthesized ssDNA pool (500 pmol) was incubated with the N protein affinity column at 4 °C for 18 h with steady shaking. After incubation, unbound DNA was washed out with 50 mM Tris–HCl buffer (containing 5 mM KCl, 100 mM NaCl, 1 mM MgCl_2_, pH 7.5) and the oligonucleotides bound in the N protein column were eluted with 10 mM Tris–HCl buffer (containing 50 mM KCl, 1.5 mM MgCl_2_, pH 8.2). The eluted reactants were ethanol-precipitated and subjected to PCR amplification (20 cycles of 95 °C for 30 s, 55 °C for 30 s, and 72 °C for 30 s, followed by a 7-min extension at 72 °C). To serve as the DNA pool for next round, PCR products were heated at 95 °C for 5 min, immediately followed by cooling on ice for 10 min right before loading to the column [17, 18]. The procedure was iterated through seven consecutive selection rounds. Additionally, to impose a more stringent condition, the incubation time was progressively reduced: 16 h (round 2), 12 h (round 3), 6 h (round 4), 3 h (round 5), 1.5 h (round 6), and 0.5 h (round 7). After the seventh selection round, DNA product was cloned into a TA vector using the T&A™ cloning kit (Yeastern Biotech, Taiwan), and transformed into *E. coli* DH5α. The plasmid DNA was isolated from individual clones using the HiYield plasmid mini kit (Yeastern Biotech, Taiwan), and the DNA sequence of aptamer#6 was determined by Mission Biotech (Taipei, Taiwan). To validate the N protein aptamers, a procedure similar to the section of SDS-PAGE and western blotting was employed for western blotting. However, in this case, 5'-biotinylated ssDNA aptamers and streptavidin-HRP conjugates were used instead of the anti-N protein antibodies and goat anti-mouse-HRP conjugates.

#### Analysis of dissociation constant (Kd)

To compare the affinity of mAbs or Apts to the SARS-CoV-2 N protein, the dissociation constant (Kd) was calculated by fitting the data to the one-site-binding model using GraphPad Prism 5.0 software (GraphPad, USA) [12]. Generally, N protein (100 µL, 0–1000 ng/mL) was coated onto the solid-phase of the microplate and incubated at 37 °C for 1 h. After washing the plate four times with PBST (350 µL per well), 200 µL of BSA-PBS (200 µL per well) was added and incubated at 37 °C. The plate was rewashed after 30 min. Next, mAb or Apt-biotin (100 µL per well; 2 ng/mL) was added and incubated at 37 °C for 30 min. After washing the plate four times, anti-mouse IgG-HRP conjugates or streptavidin-HRP conjugates (100 µL per well; 1 µg/mL) was added and incubated at 37 °C for 30 min, respectively. The plate was washed again with PBST, and 100 µL of TMB substrate solution was added. After allowing the color to develop for 15 min, the reaction was stopped by adding 1 N HCl (100 µL per well). The absorbance value was read using an ELISA reader (Vmax, Molecular Devices Co., USA) at 450–650 nm.

#### Prediction of secondary structure and docking site

The procedure of amplification variable-region genes of monoclonal antibody was outlined in supplementary section (Amplification variable-region genes of monoclonal antibody). The secondary structures of mAb-6F6E11 and selected ssDNA aptamers were predicted using the SWISS-MODEL web server (https://swissmodel.expasy.org/) [20, 21] and RNAfold web server (http://rna.tbi.univie.ac.at/cgi-bin/RNAWebSuite/RNAfold.cgi) [22], respectively, while the docking sites of the mAb-6F6E11 or aptamers to the SARS-CoV-2 N protein were predicted using the HDOCK web server (http://hdock.phys.hust.edu.cn) [23].

### Conjugation of SARS-CoV-2 N protein aptamer to different proteins

#### Coupling the aptamer to ovalbumin (OVA)

The N protein aptamers were conjugated to OVA (ovalbumin) using glutaraldehyde with slight modification [24]. To activate the amine groups on OVA, 20 µL of 25% glutaraldehyde was added to the OVA solution (1.6 mg dissolved in 400 µL PB buffer), and the mixture was incubated overnight at room temperature with continuous stirring. Subsequently, the reaction mixture was dialyzed against 0.1 M carbonate buffer (pH 9.5) for one day. Next, the apt-NH_2_ solutions (0.2 mg in 0.1 M carbonate buffer, pH 9.5; synthesized by Mission Biotech, Taiwan) were added to the activated OVA mixture. Finally, 300 µL of 1 M lysine solution was added to quench the reaction, followed by dialysis with 0.01 M PBS to remove any unreacted components.

#### Conjugation of the aptamer to horseradish peroxidase (HRP)

The aptamer was covalently coupled to HRP using sodium periodate [25]. First, 1.6 mg HRP was dissolved in 400 µL deionized water and 100 µL of 100 mM sodium periodate solution was added. After incubating the mixture at room temperature for 30 min, the reactant dialyzed overnight at 4 °C against sodium acetate buffer (1 mM, pH 4.4). After dialysis, 10 µL of carbonate buffer (0.2 M, pH 9.5) was added to the HRP reactant and mixed with 300 µL of apt-NH_2_ solution (0.2 mg in 0.1 M carbonate buffer, pH 9.5). After incubating the mixture at room temperature for 2 h, 100 µL of a NaBH_4_ solution (4 mg/mL) was added to the reactant for the terminal reaction. The mixture was then incubated at room temperature for 2 h and dialyzed with 0.01 M PBS.

### Development of enzyme-linked aptamer-antibody sandwich assay (ELAAA)

The ELAAA procedure was carried out as follows. First, monoclonal antibodies against SARS-CoV-2 N protein (100 µL) were added to each well of the plate and incubated at 37 °C for 1 h. The plate was then washed four times with PBST and blocked with 200 µL of BSA-PBS for 30 min at 37 °C. Prior to the competition step, the plate was rewashed with PBS-Tween. Next, a mixture of N protein standards (50 µL, 0.1–500 ng/mL) and Apt-HRP conjugates (50 µL, diluted with BSA-PBST, 0.1% BSA and 0.05% Tween in 0.01 M PBS) was added to each well and incubated at 37 °C for 30 min. Next, the plate was washed again and 100 µL of TMB substrate solution was added, followed by incubation at room temperature for 20 min in the dark. The reaction was terminated by adding 100 µL of 1 N HCl and the absorbance value was measured at 450–650 nm using an ELISA reader (Fig. 1A).

To ensure long-term storage, the microtiter plate was vacuum-sealed and stored at 4 °C after coating each well with the mAbs and blocking with a solution containing 0.1% BSA and 0.5% carbohydrate in 0.01 M PBS [26]. Additionally, the Apt-HRP conjugate solution was diluted in a stabilized buffer (0.01 M PBS containing antibacterial agents) [26] and stored at 4 °C.

### Establishment of hybrid lateral flow strip (hybrid-LFS)

#### Synthesis of gold nanoparticles

Following a previously reported protocol, the 40 nm gold nanoparticles (GNPs) were synthesized using trisodium citrate as a reducing agent [27]. Briefly, a mixture of 1 mM gold(III) chloride trihydrate and 0.1 nM mPEG-carboxymethyl was heated with constant stirring, and 2.5 mL of 34 mM trisodium citrate was added while boiling. After 1 min of continuous boiling, the mixture was cooled to room temperature to obtain carboxylic acid-functionalized 40 nm GNPs, which were used to prepare mAb-GNP probes.

#### Preparation of mAb-gold nanoparticle (mAb-GNP) Conjugates

To prepare mAb-GNP conjugates as probes for the hybrid-lateral flow strip, a slightly modified conjugation method was used, as reported previously [27]. Specifically, 200 μg of purified N protein mAb in 0.1 mL of 2 mM borax buffer was added to 2 mL of the gold nanoparticle solution, followed by the dropwise addition of 1.5 μL of 10% EDC solution and 0.8 μL of 10% N-hydroxysuccinimide (NHS) solution with constant stirring at room temperature for 1 h. Next, 220 μL of 10% BSA solution was added to block the unbound surface of GNPs. After incubation at room temperature for 30 min, the reaction mixture was centrifuged at 13,000 rpm for 30 min at 4 °C, and the resulting pellet was dissolved in 0.2 mL of 20 mM Tris buffer (pH 8.0) containing 1% BSA and 0.1% sodium azide. The mAb-GNP probe was stored at 4 °C for further use.

#### Assembly of hybrid lateral flow strip

The hybrid-LFS comprises four components: an absorbent pad, a nitrocellulose (NC) membrane with a control zone and a test zone (as depicted in Fig. 1B), a release pad, and a sample pad. To begin, the release pad was pre-treated with a solution of 10 mM sodium borax, 2% BSA, 4% sucrose, and 0.01% sodium azide and then dried overnight at 37 °C. The N protein mAb-GNP (8 μL) were then sprayed onto the release pad. The test and control zones on the NC membrane were drawn with Apt-OVA and goat anti-mouse secondary antibodies, respectively. Next, the NC membrane and release pad were dried at 37 °C for 30 min. The hybrid-LFS was then assembled according to the procedure described by Wu et al. (2022) (Fig. 1B) [26]. Finally, the assembled hybrid-LFS is placed in a cassette and vacuum-packaged for a long-term storage.

#### Determination of the visual detection limit

Following assembly, SARS-CoV-2 N protein standard solutions (0.1 – 500 ng/mL) were added to the sample zone, and the solutions moved along the membrane by capillary action. The visual results on the hybrid-LFSs were assessed after 10 min, and the color density of the red line was read using a strip scan reader (Taiwan Advance Bio-Pharmaceutical Inc., Taiwan).

### Stability

The stability testing procedures for the hybrid ELAAA and hybrid-LFS were conducted in accordance with a previous report [26]. To evaluate the stability of the mAb-coated plate, N protein standard solution and Apt-HRP solution, they were stored at 4 °C for 1 year, and the absorbance value was monitored at 3, 6, and 12 months. In addition, the hybrid-LFS, after completing the test and control line drawing and assembly, was vacuum-packaged and stored at room temperature for 1 year. The strength of the test line was analyzed at 12 month to assess the stability of the hybrid-LFSs.

### Nasopharyngeal sample analysis

#### Sample preparation

The 300 µL extraction solution (100 mM Tris–HCl, pH 9.0) used for the sample preparation contained 100 mM NaCl, 1% Tween 20, and 0.1% sodium azide, as described in a previous report (Patriquin et al., 2021). To prepare the nasopharyngeal sample, a nasopharyngeal swab (COPAN Diagnostics Inc., USA) was inserted into the nasal cavity about 2 – 3 cm and rotated 4 – 5 times along the inner wall of the nasal cavity. The swab was then dipped into the extraction solution and spun 3 – 5 times to obtain the sample extract solutions. The extract solutions were analyzed using hybrid-LFS. In addition, the negative sample extract solutions were spiked N protein before determining through the hybrid ELAAA.

#### Determination of SARS-CoV-2 N protein in spiked nasopharyngeal samples using hybrid ELAAA

The hybrid ELAAA package was opened and allowed to equilibrate to room temperature. Then, 50 µL of the diluted sample extraction solution, prepared using BSA-PBST or a SARS-CoV-2 N protein standard with concentrations ranging from 0.001 to 500 ng/mL, along with 50 µL of Apt-HRP conjugate solution, were added to the microplate well. The reaction was allowed to proceed for 30 min at 37 °C, followed by washing with PBST and the addition of 100 µL of TMB substrate. After a 15 min incubation period, 100 µL of 1 N HCl was added to stop the reaction, and the absorbance value was measured using a Vmax reader at 450 – 650 nm. The SARS-CoV-2 N protein levels in the sample extracts were determined by calculating them from the calibration curve.

#### Detection of SARS-CoV-2 N protein levels in nasopharyngeal samples using hybrid-LFS

For the analysis of real sample extract solutions, 120 µL (equivalent to three drops) of the extraction solution from 5 infected individuals and 10 healthy volunteers were added to the sample zone of the hybrid-LFS. The sample solution was then allowed to flow through the test and control lines via capillary action. The results of hybrid-LFSs were obtained and photographs were taken by volunteer themselves after a 10-min reaction period.

## Results

### Expression of SARS-CoV-2 nucleocapsid protein

**Fig. 2:**
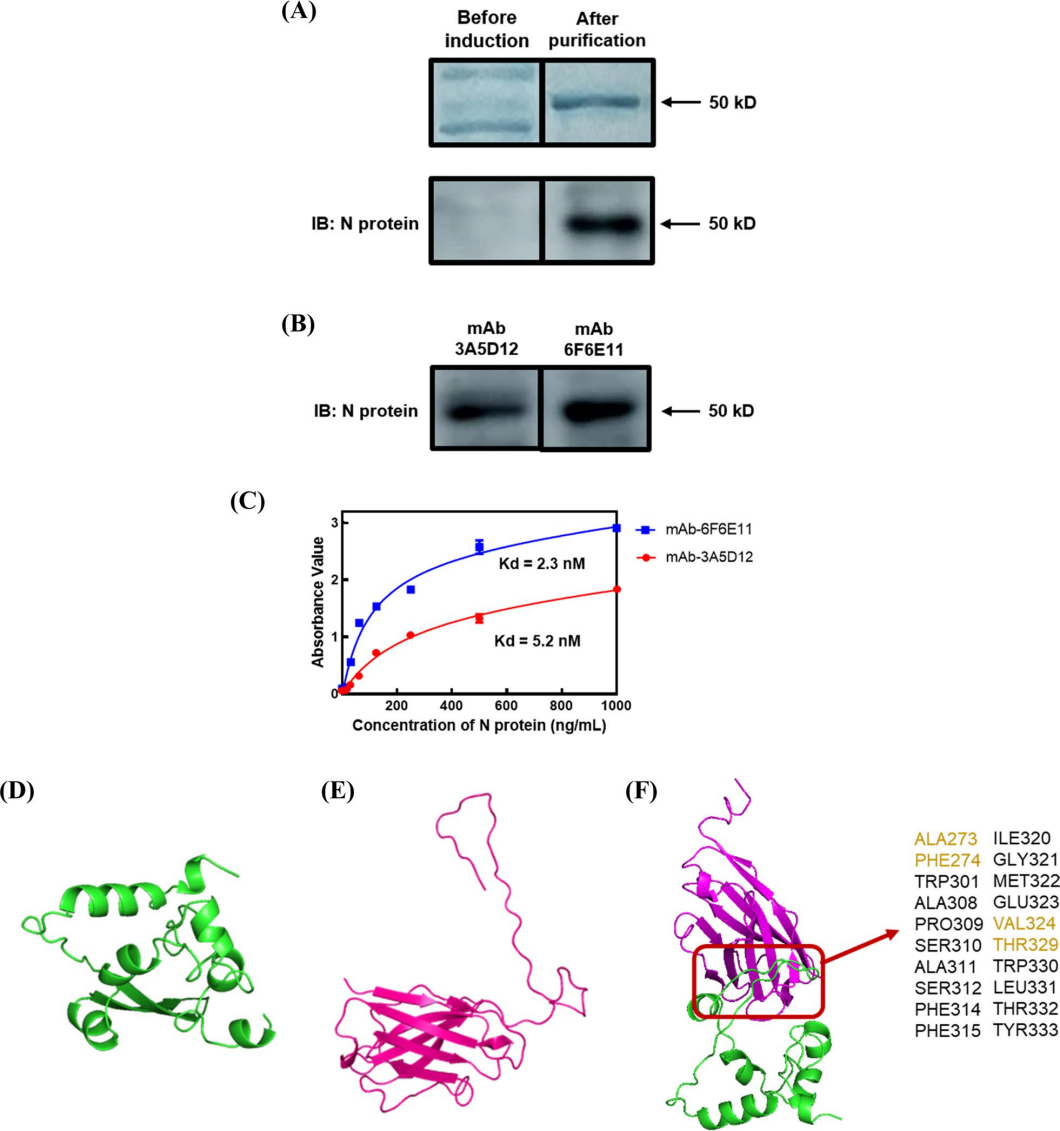
Characterization of SARS-CoV-2 nucleocapsid protein with monoclonal antibodies specific to the nucleocapsid protein. **A** SDS-PAGE analysis of N expression protein (upper panel); western blotting analysis of N expression protein using mouse serum (bottom panel). **B** Western blotting analysis of N protein using mAbs produced in this study. **C** Analysis of the binding affinity (Kd value) of monoclonal antibody-3A5D12 ( ) and 6F6E11 ( ). The results represent the mean ± SD of absorbance value obtained from three independent replicates. **D** Three-dimensional structure prediction of SARS-CoV-2 nucleocapsid protein. **E** Three-dimensional structure prediction of the monoclonal antibody-6F6E11. **F** Docking site prediction between N protein and mAb-6F6E11

To generate immune antigens for the production of aptamers and monoclonal antibodies, the SARS-CoV-2 N protein was expressed and purified from bacterial cell lysates. The N protein plasmid-containing bacterial cells were cultured at 37 °C for 18 h while induced with 0.5 mM IPTG. The recombinant protein was then made water-soluble and refolded using 8 M Urea and ammonium sulfate precipitation, respectively. Subsequently, the recombinant protein was purified using a Ni–NTA column. Approximately 2 mg of the recombinant protein was obtained from 1 L of bacterial cell culture. The expression and purification process resulted in a high level of recombinant protein, as observed by 10% SDS-PAGE. The molecular weight of the recombinant protein, approximately 50 kDa, matched that of the SARS-CoV-2 N protein (Fig. 2A, upper panel). To validate the expression of the SARS-CoV-2 N protein, western blotting was conducted using an anti-N protein antibody acquired from LEADGENE Biomedical Inc. (Tainan, Taiwan). Additional file 1: Fig. S1 demonstrates the specific identification of the recombinant protein as the SARS-CoV-2 N protein. Therefore, these recombinant N proteins were utilized in subsequent experiments.

### Production and characterization of N protein monoclonal antibodies

To obtain monoclonal antibodies specific to the SARS-CoV-2 N protein, BalB/c mice were immunized with the N expression protein. The mice sera were collected and analyzed for the presence of specific antibodies against the N protein. The western blotting results using mice serum confirmed the presence of specific N protein antibodies (Fig. 2A, bottom panel). The highest specificity of the serum was observed at 8 weeks of immunization (data not shown). In order to generate hybridoma cells that secrete monoclonal antibodies targeting the N protein, the mouse spleen was fused with a myeloma cell line for hybridoma screening and antibody secretion. The screening process involved the utilization of iELISA and western blotting techniques. Out of the 900 culture wells tested, two wells (3A5 and 6F6) demonstrated a robust positive signal indicating the presence of colonies secreting antibodies with higher specificity against the N protein. These colonies were isolated from the fusion well and subjected to the limiting dilution step. From this step, the positive clones 3A5D12 and 6F6E11 were selected to produce culture supernatants against the N protein (Fig. 2B). In order to determine the antibodies affinity to the N protein. The dissociation constant (Kd) values of mAb-3A5D12 and 6F6E11 were calculated as 5.2 nM and 2.3 nM, respectively (Fig. 2C). Consequently, mAb-6F6E11, which demonstrated high specificity to the SARS-CoV-2 N protein, was chosen for further development of hybrid ELAAA and hybrid-LFS. The sequence of the N protein and DNA sequences of ScFv were subjected to structure prediction using the SWISS-MODEL. SWISS-MODEL is an automated web-based server used in bioinformatics and structural biology to predict the 3D structure of a target protein based on homology modeling principles [20, 21]. The Fig. 2D and Fig. 2E provided visual representations of the predicted structures of the N protein and ScFv, respectively. Docking site prediction analysis provided an estimation of the approximate binding site of mAb-6F6E11 to the N protein (Fig. 2F).

### Generation and characterization of aptamers specific to N protein

**Fig. 3:**
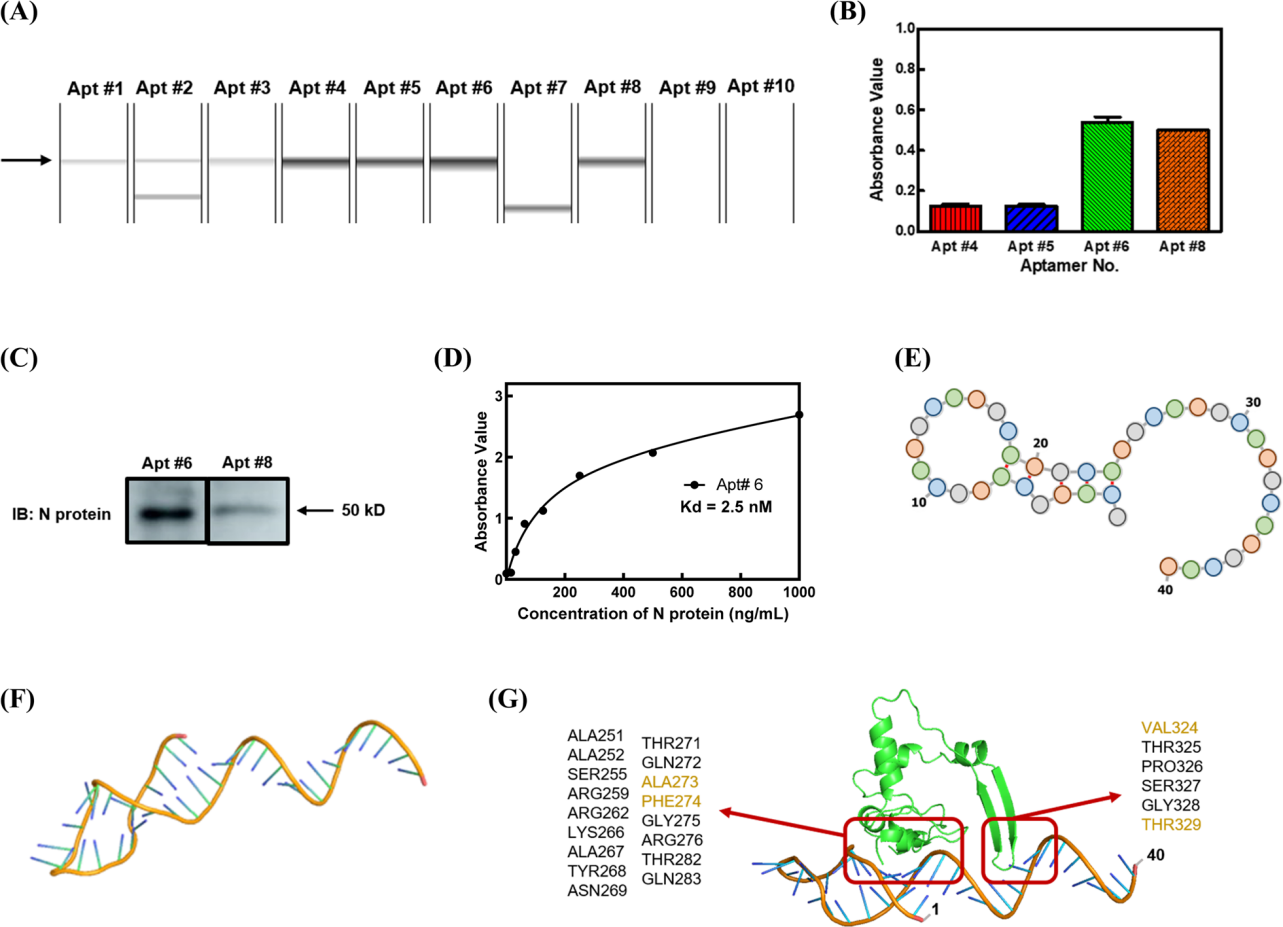
Characterization of the SARS-CoV-2 N protein aptamer. **A** Selection of aptamer after SELEX using capillary electrophoresis. **B** Affinity analysis of aptamer to N protein using iELISA. **C** Western blotting analysis of Apt #6 and Apt #8 affinity to N protein. **D** Analysis of the binding affinity (Kd value) of Apt #6. The results represent the mean ± SD of absorbance value obtained from three independent replicates. **E** Secondary structure prediction of Apt #6. **F** Three-dimensional structure prediction of the Apt#6 **G** Docking site prediction between N protein and Apt #6

The N protein was initially conjugated to the CarboxyLink™ Coupling gel using the carbodiimide method. The conjugation rate of the N protein affinity column was determined to be 49% using the Bio-Rad protein assay system (data not shown). Subsequently, ssDNA aptamers targeting the N protein were selected through SELEX using the N protein affinity column. After 7 rounds of selection, the selected DNAs were cloned into a TA vector and transformed into *E. coli* DH5α. Plasmid DNA containing the aptamers was isolated from individual clones. Figure 3A displays the 10 candidate aptamers that exhibited affinity to the N protein. However, Apt #9 and Apt #10 were excluded as they did not show any DNA band upon capillary electrophoresis (Fig. 3A). Apt #7 was also excluded due to incorrect DNA size (Fig. 3A). Additionally, Apt #2 and Apt #3 were excluded from further consideration due to the presence of non-specific products with incorrect DNA sizes, as depicted in Fig. 3A. Figure 3B demonstrates the higher affinity of Apt #6 and Apt #8 towards the N protein. This observation was further validated through western blotting, which revealed that Apt #6 exhibited excellent affinity to the N protein (Fig. 3C). The dissociation constant (Kd) of Apt #6 was determined to be 2.5 nM (Fig. 3D). Figure 3E provides the DNA sequence interpretation and the predicted secondary structure. Based on these findings, Apt #6 was chosen for the development of hybrid ELAAA and hybrid-LFS. The sequence of the N protein and Apt #6 were utilized for structure prediction using the SWISS-MODEL and RNAfold web servers. The obtained results provided visual representations of the predicted structures of the N protein and Apt #6 (Fig. 2D & 3F). Docking site prediction analysis revealed the precise binding sites of Apt #6 to the N protein (Fig. 3G). The binding locations of the mAb-6F6E11 or Apt #6 to the N protein exhibited significant differences, indicating unique binding mechanisms and interactions between these two molecules and the N protein (Figs. 2F & 3G).

### Establishment of hybrid ELAAA and hybrid-LFS

#### Development of hybrid ELAAA

**Fig. 4:**
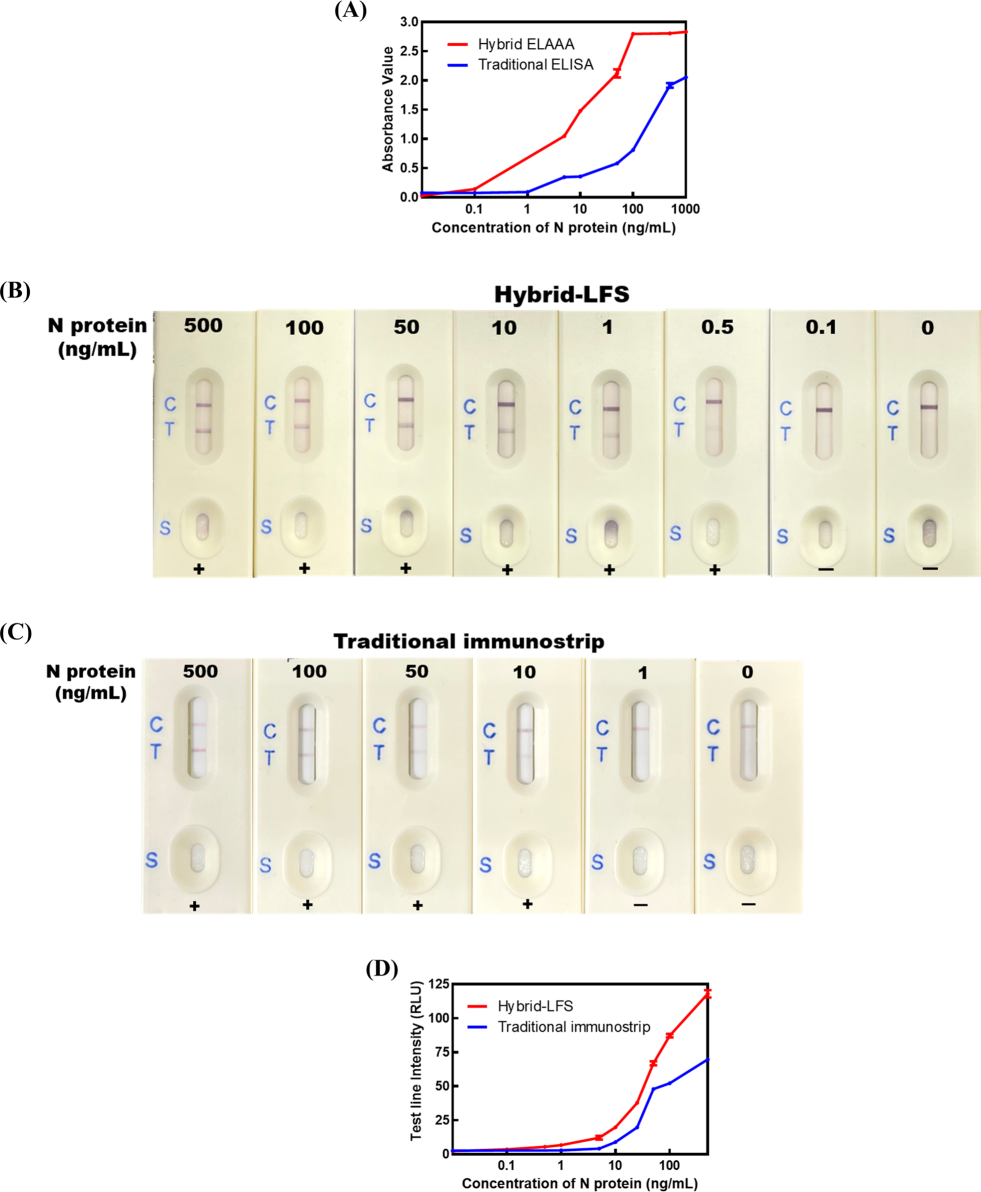
Characterization of the SARS-CoV-2 enzyme-linked aptamer-antibody sandwich assay and hybrid lateral flow strip. **A** Standard curves of the hybrid ELAAA ( ) and traditional double antibodies-based ELISA ( ) for the N protein standard. The results represent the mean ± SD of absorbance value obtained from three independent replicates. **B** The visual detection limit of the hybrid-LFS for the N protein standard in the extraction solution. **C** The visual detection limit of the traditional double antibodies-based immunostrip for the N protein standard in the extraction solution. **D** Standard curves of the test line intensity on the hybrid-LFS ( ) and traditional antibodies-based immunostrip ( ). The results represent the mean ± SD of test line intensity (RLU) obtained from three independent replicates

In order to rapidly and accurately detect the presence of the N protein in nasopharyngeal samples, a hybrid ELAAA utilizing aptamer and monoclonal antibody was developed. The principle of the hybrid ELAAA is illustrated in Fig. 1A Initially, the solid plate was coated with mAb-6F6E11. Subsequently, the nasopharyngeal sample and Apt-HRP conjugates were introduced to the plate, facilitating simultaneous binding of the Apt-HRP conjugates and mAb-6F6E11 to distinct sites on the N protein. This binding interaction enables the Apt-HRP conjugates to generate a color reaction when the TMB substrate is added, indicating the level of N protein in the nasopharyngeal samples. The hybrid ELAAA conditions were optimized in this study, resulting in a detection limit of 0.1 ng/mL and an assay procedure time of 40 – 50 min. Comparative analysis with the traditional double antibodies-based sandwich ELISA developed in our laboratory demonstrated a sensitivity improvement of over 50-fold for the hybrid ELAAA, as the detection limit of the traditional method was 5 ng/mL of N protein standard (Fig. 4A). To assess the long-term stability of the hybrid sandwich ELAAA during storage, a stability analysis was performed. A blocking buffer comprising 0.5% carbohydrate and 0.1% BSA was applied to protect the N protein monoclonal antibodies coated on the microtiter plate. Additional file 1: Fig. S2A presents the stability analysis of the hybrid sandwich ELAAA stored at 4 °C, revealing a 60% decrease in absorbance value after one year of storage.

#### Development of hybrid-LFS

The hybrid-LFS offers a visually interpretable and more rapid alternative to the hybrid ELAAA. The principle of the hybrid-LFS is illustrated in Fig. 1B Since the N protein first reacts with the mAb-GNP, and then reacts with the Apt-OVA, when the concentration of N protein in the sample exceeds a certain threshold, the N protein binds successively to mAb-GNP conjugates in the release pad and Apt-OVA on the test line. This results in the appearance of a red line on both the test and control lines of the hybrid-LFS, indicating a positive result ( +). The control line, which contains the goat anti-mouse secondary antibody, serves as a control to confirm whether the hybrid-LFS assay ran correctly. The control line should always appear, irrespective of the presence or absence of N protein; its absence indicates poor manufacturing or improper usage of the hybrid-LFS. The hybrid-LFS conditions were optimized in this study. The optimized hybrid-LFS were used to analyze N protein standards with concentrations of 0, 0.1, 0.5, 1, 10, 50, 100 and 500 ng/mL. In Fig. 4B, 120 µL of each N protein standard was added to the sample zone to initiate capillary motion. When an N protein concentration of 0.1 ng/mL, no red line was observed on the test line, indicating that the visual detection limit of the hybrid-LFS was between 0.1 – 0.5 ng/mL (Fig. 4B). Additionally, the color density of the test line was less than 5 RLU (relative light units) (Fig. 4D). In comparison, the visual detection limit of the traditional double antibody immunostrip for N protein developed in our laboratory was between 1 – 10 ng/mL, indicating a more than tenfold improvement in sensitivity for the hybrid-LFS (Fig. 4C). The intensity of the test line was measured by a hybrid-LFS scan reader and the values were used to establish the standard curves in Fig. 4D. For commercial production of the hybrid-LFSs, it is crucial to assess their storage stability. Therefore, vacuum-packed hybrid-LFSs were stored at room temperature, and the sensitivity and strength of the test lines were evaluated at 12 month. The results demonstrated a slight decrease in color intensity of the test lines at an N protein concentration of 0.5 ng/mL. However, the visual detection limit did not deteriorate after one year of storage (Additional file 1: Fig. S2B & C). Furthermore, the intensity of the control lines did not decrease either. Therefore, the hybrid-LFSs developed in this study can be stored for at least one year.

## Sample analysis

### Analysis of N protein levels in spiked nasopharyngeal samples using hybrid ELAAA and hybrid-LFS

The effectiveness of the hybrid ELAAA in analyzing the concentration of N protein in nasopharyngeal samples was assessed. Initially, all samples were found to be free of N protein, demonstrating the absence of false positives (Table 1). Subsequently, a recovery study was conducted to evaluate the accuracy of the hybrid ELAAA in analyzing spiked nasopharyngeal samples. For the negative samples spiked at concentrations ranging from 0.5 to 1000 ng/mL, the analytical recovery rates of N protein ranged from 80 to 99%, with coefficients of variation (CV) values ranging from 0.5% to 9.5% (Table 1). If the SARS-CoV-2 N protein concentration surpasses 1000 ng/mL and precise quantitation is sought, the nasopharyngeal samples must be diluted 5 folds to fit in the standard curve of hybrid-ELAAA. The overall average analytical recovery rate for all the spiked nasopharyngeal samples was determined to be 92% (CV, 4.4%) (Table 1).

**Table 1:** Analysis of uninfected and spiked nasopharyngeal samples using hybrid ELAAA and hybrid-LFS

No.^a^	Spike N protein (ng/mL)	ELAAA (ng/mL)^b^	CV (%)^c^	Recovery (%)	Hybrid-LFS
N_1_ – N_10_	0	N.D^d^			―
N_1_-S	1000	990 ± 22.2	2.2	99	+
N_2_-S		917 ± 50.4	5.5	92	+
N_3_-S	500	461 ± 20.8	4.5	92	+
N_4_-S		487 ± 43.0	8.8	97	+
N_5_-S	50	40 ± 0.4	1.0	80	+
N_6_-S		43 ± 0.2	0.5	86	+
N_7_-S	1	0.95 ± 0.09	9.5	95	+
N_8_-S		0.96 ± 0.03	3.1	96	+
N_9_-S	0.5	0.47 ± 0.02	4.3	94	+
N_10_-S		0.44 ± 0.02	4.5	88	+
Overall			4.4	92	

Each sample was analyzed in three repeats; ^a^N_1_ – N_10_ corresponds to 10 volunteers, while N_1_-S –N_10_-S indicates their respective spiked samples; ^b^The ELAAA results are presented as mean ± SD; ^c^The Coefficient of Variation (CV) is calculated using the following formula: $\frac{SD}{Mean}\times 100\%$; ^d^N.D, the measured N protein concentrations were all below the detection limit of the hybrid ELAAA

### Analysis of N protein levels in infected nasopharyngeal samples using hybrid-LFS

**Fig. 5:**
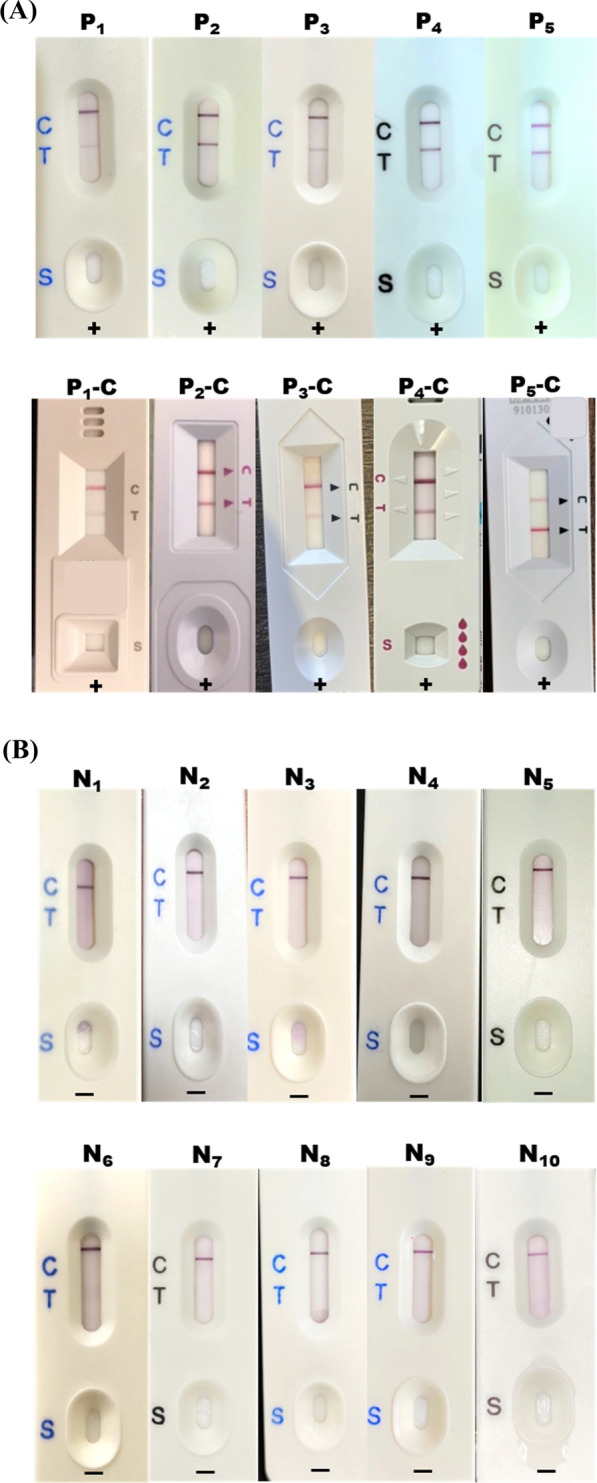
Analysis of SARS-CoV-2 N protein levels in nasopharyngeal samples using hybrid-LFS. **A** Analysis of 5 positive infected samples using hybrid-LFS (upper panel) and commercial antigen rapid test strip (lower panel). **B** Analysis of 10 negative samples using hybrid-LFS. Each hybrid-LFS analysis was processed by the volunteers themselves

The hybrid-LFSs were employed to perform qualitative analysis of the N protein in 5 nasopharyngeal samples from infected individuals, 10 samples from healthy volunteers, and an additional 10 nasopharyngeal samples spiked with the N protein. The analysis using the hybrid-LFSs was conducted by the volunteers themselves, who also captured photographs of the results. In the analysis, the nasopharyngeal samples extracts were applied to the sample zone of the hybrid-LFS to determine the presence of N protein. The results revealed that samples P_1_, P_2_, P_3_, P_4_, and P_5_ displayed positive results, with the presence of two red lines (C and T), confirming the presence of N protein (Fig. 5A, upper panel). For comparison, the 5 infected individuals also utilized a commercial antigen rapid test strip at the same time (Fig. 5A, bottom panel), which further highlighted the high accuracy of the hybrid-LFS in detecting N protein. Conversely, samples N_1_, N_2_, N_3_, N_4_, N_5_, N_6_, N_7_, N_8_, N_9_, and N_10_ showed negative results, with only one red control line (Fig. 5B), indicating the absence of N protein. Furthermore, the hybrid-LFS exhibited consistent results with the hybrid ELAAA when analyzing the 10 spiked nasopharyngeal samples (Table 1), thereby reinforcing the reliability of the hybrid aptamer-antibody approach.

## Discussions

The SARS-CoV-2 nucleocapsid protein was expressed and utilized as a target of coupling gel for aptamer generation and used as an immunogen for the production of monoclonal antibodies. We evaluated three crucial factors related to expression efficiency, the yield of the expressed protein, and induction time. The concentration of IPTG used for induction (0.05, 0.1, and 0.5 mM) was assessed for its effect on expression efficiency. The induction temperature (25 and 37 °C) was investigated for its impact on the yield of the expressed protein. Additionally, the induction time (2, 6, 12, and 18 h) was tested to determine its influence on the yield of the expressed protein. These experiments allowed us to identify the optimal conditions for protein expression in our system. The results indicated that the optimal conditions for obtaining the highest expression of the N protein in non-water-soluble inclusion bodies involved inducing bacterial cells containing the N protein plasmid with 0.5 mM IPTG at 25 °C for 18 h (data not shown). Subsequently, inspired by previous reports, the N expression protein present in the inclusion bodies was denatured using a high concentration of urea (8 M) to render it water-soluble [28]. Refolding of the N expression protein was then performed using the ammonium sulfate precipitation method, which offered the advantage of excluding proteases [29, 30]. Finally, purification of the N expression protein was accomplished using the Ni–NTA column. Following purification, the quantity of the N expression protein was measured to be 2 mg, which aligns with the findings reported in previous studies [31]. Furthermore, the identity of the expression protein as SARS-CoV-2 N protein was confirmed through additional analysis using SDS-PAGE. (Fig. 2A, upper panel) These analyses provided further validation of the correctness of the protein. The N protein was utilized for the immunization of mice, followed by the screening of hybridoma cells to identify monoclonal antibodies specific to the N protein using iELISA. Out of the 900 culture wells analyzed, two wells, namely 3A5 and 6F6, exhibited a robust positive signal, indicating the presence of colonies secreting antibodies with the highest specificity against the N protein. Further characterization of the Kd of mAb-3A5D12 and 6F6E11 revealed that mAb-6F6E11 exhibited superior specificity (Fig. 2C). Therefore, mAb-6F6E11 was selected for the subsequent establishment of the hybrid ELAAA and hybrid-LFS. Moreover, a comparison was conducted between the developed monoclonal antibodies and commercial SARS-CoV-2 N protein antibodies. All antibodies were diluted to the same concentration, and the microplate was pre-coated with serially diluted N protein. By determining the N protein dilution factor at an absorbance value of 1.5 after a 15-min TMB substrate reaction, the titration of antibodies was determined. The data demonstrated that mAb-6F6E11 exhibited the highest titration among the tested antibodies (Additional file 1: Table S1). This finding further highlights the superior performance of mAb-6F6E11 and underscores its potential as a valuable tool for SARS-CoV-2 N protein detection in diagnostic applications.

The N protein was also conjugated to the CarboxyLink™ coupling gel using the carbodiimide method, creating an N protein affinity column that was utilized for SELEX to generate high-affinity N protein aptamers. During the SELEX procedure, starting with long incubation times in the very early rounds of SELEX allowed us to include a broad spectrum of aptamer sequences, including those with moderate to weak binding affinities. As the SELEX process advanced further through the following rounds, the incubation times gradually reduced to favor the enrichment of aptamer sequences with higher binding affinities. Finally, this approach helps us to optimize the identification of aptamer with a high specificity and faster binding kinetics for the development of a quick assay. Moreover, PCR plays a crucial role in generating a DNA library for the subsequent SELEX rounds. The optimization of PCR parameters is essential to ensure successful amplification without the formation of primer dimers or insufficient product DNA concentration. Real-time PCR analysis was performed to determine the optimal number of PCR cycles for DNA amplification, and the results indicated that 18 – 20 cycles of PCR provided the best amplification (data not shown). Therefore, 20 cycles of PCR amplification were selected to ensure an adequate amount of DNA for the subsequent SELEX rounds. The concentration of primers used in PCR is a critical factor that affects annealing efficiency and the occurrence of mispriming and nonspecific amplification. Therefore, careful control of primer quantity is necessary during the SELEX procedure. The 0.25 µM of primers were selected to perform PCR in this study. Additionally, the tRNA as a carrier for precipitation and visibility of the DNA pellet was mentioned in the previous article [32]. To improve the SELEX procedure, additional tRNA was added to increase DNA precipitation yield in this study. Furthermore, the reaction time between ssDNA and the N protein affinity column was optimized to increase the probability of selecting high-affinity aptamers. It was observed that reducing the reaction time yielded better selection results compared to increasing the salt concentration in the elution solution of SELEX (data not shown). After 7 rounds of SELEX, Apt #6 and Apt #8 were selected as potential high-affinity aptamers (Fig. 3B). Western blotting analysis revealed that Apt #6 exhibited a higher affinity to the N protein (Fig. 3C). The Kd of Apt #6 was further determined to be 2.5 nM (Fig. 3D), demonstrating an improved affinity compared to the previously reported value of 4.5 nM [33]. These findings highlight the successful generation of high-affinity N protein aptamers as a valuable tool for SARS-CoV-2 N protein detection in diagnostic applications.

In the previous double aptamers sandwich analysis procedure, aptamers are typically coated onto a microplate, and the target is added subsequently. Then, another aptamer conjugated with biotin is introduced, followed by the addition of streptavidin-HRP and TMB substrate to obtain the results. However, the use of streptavidin-HRP is costly and requires a large amount for single-use, which limits the development of aptamer sandwich assays. In this study, we followed the approach described by Li et al. [34]. The 3' end of the aptamer was modified with an amine group (NH_2_) and conjugated with HRP using sodium periodate. Consequently, the step of aptamer-biotin and streptavidin-HRP reaction was eliminated, and the result could be directly obtained by using TMB substrate after the Apt-HRP reacted with the target. The use of Apt-HRP offered advantages such as reduced detection costs and shorter detection times. To minimize assay background values in the non-competitive hybrid ELAAA, several measures were taken to reduce the protein adsorbed on the microplate. When the Apt-HRP conjugates bind to the sample on the microplate instead of binding to the sample coupled to the mAb, leading to a high background value. To address this issue, this study followed the protocols described by Apollonio et al. and Wang et al. by using BSA-PBST as the assay diluent to minimize background values [35, 36]. Additionally, the hybrid ELAAA was optimized by adjusting the assay procedure and the reaction time of the sample with Apt-HRP conjugate and TMB substrate chromogenic reactions. The optimized assay involved adding the sample or standard together with Apt-HRP into the microplate to react with the mAb that was already coated on the microplate, rather than first allowing the sample to react with the mAb and then adding Apt-HRP. Furthermore, the reaction time was reduced from 1 h to 15 min, and the TMB substrate chromogenic reaction time was set at 15 min. After the optimization, the hybrid ELAAA had a detection limit of 0.1 ng/mL with the assay procedure of 40 – 50 min (Fig. 4A). In addition, a comparison with commercial ELISA kits for SARS-CoV-2 antigen detection was conducted. Additional file 1: Table S2 demonstrates that the detection limits of the commercial ELISA kits ranged from 0.07 to 0.78 ng/mL, with assay procedure times ranging from 4 to 5 h. The detection limit of the hybrid ELAAA was as low as that of the commercial ELISA kits, the assay procedure time was shorter than all the commercial kits (Additional file 1: Table S2). Therefore, the results of the hybrid ELAAA could be obtained more quickly, contributing to faster disease control and prevention.

Many articles have discussed the conjugation of aptamers with gold nanoparticles, often involving the affinity coupling of aptamer-biotin and streptavidin [37–39]. However, in order to couple streptavidin to gold nanoparticles, it is necessary to first couple streptavidin to the gold nanoparticles before aptamer-biotin can be conjugated to them. Previous tests have shown that the conjugation of streptavidin with gold nanoparticles requires the use of high concentrations of streptavidin, which increases the production cost of the hybrid-LFS. To reduce production costs, in this study, the aptamer was conjugated to ovalbumin (OVA) using glutaraldehyde. The Apt-OVA conjugates could then be adsorbed onto the NC membrane as the test line, while mAb-6F6E11 was adsorbed onto gold nanoparticles as the detection probe. We carefully regulated three critical factors to optimize the hybrid-LFA. Initially, 20 nm amine-functionalized gold nanoparticles were utilized to conjugate with 150 µg of antibodies. The investigation focused on assessing the influence of varying pH levels (pH 5.5, 6.5, 7.5, and 8.5) within the gold nanoparticle-antibody conjugation buffer. The objective was to determine the impact of pH on the adsorption efficiency between gold nanoparticles and antibodies, as illustrated in Additional file 1: Figure S3A. Subsequently, employing a conjugation buffer set at pH 5.5 and using 150 µg of antibodies for the gold nanoparticle conjugation process, an exploration was conducted with diverse particle sizes (15, 20, 30, and 40 nm) of gold nanoparticles. The aim was to identify the optimal nanoparticle size that would yield maximum antibody adsorption on the nanoparticle surface. These findings are presented in Additional file 1: Figure S3B. Finally, utilizing 40 nm amine-functionalized gold nanoparticles along with a conjugation buffer set at pH 5.5, an examination was performed concerning the adsorption of varying quantities of antibodies (50, 100, 150, 200, 250 μg) onto the surface of gold nanoparticles. The intention was to get a balance between performance and cost-effectiveness, as depicted in Additional file 1: Figure S3C. After optimization, it was found that a pH value of 5.5 for the conjugation buffer, gold nanoparticles with a particle size of 40 nm, and 200 μg of mAb adsorbed provided the best cost-effectiveness and test line intensity. The visual detection limit of hybrid-LFS was analyzed to be 0.1 – 0.5 ng/mL N protein (Fig. 4B). Furthermore, a comparison with several commercial antigen rapid test strips for SARS-CoV-2 detection was conducted. When the N protein standards were dissolved in the extraction solution used in this study, the detection limits of the commercial antigen rapid test strips were observed to range from 0.5 to 500 ng/mL. These results were worse than the detection limits achieved in our study, which ranged from 0.1 to 0.5 ng/mL (Additional file 1: Table S3). Consequently, the hybrid-LFS developed in this research offers significant advantages in terms of low cost and high sensitivity, thus enabling faster disease control and prevention.

To prevent false-positive detections, this study referred to a previous report that used glass fiber for the sample pad [40]. Additionally, the release pad was pre-treated with a solution of 10 mM sodium borax containing 2% BSA, 4% sucrose, and 0.01% sodium azide to prevent false positives. Moreover, Patriquin et al. mentioned that increasing the salt concentration in the extraction solution could improve false-positive detection [40]. Higher salt concentration creates a more challenging condition for antibody-antigen coupling, reducing non-specific binding and improving false-positive analysis. Patriquin et al. also suggested that the pH value of the extraction solution should be more alkaline than the mAb-GNP conjugation buffer to prevent binding of gold nanoparticles to the Apt-OVA conjugates on the test line during the analysis procedure [40]. Therefore, a solution of 100 mM Tris–HCl (pH 9.0) containing 100 mM NaCl, 1% Tween 20, and 0.1% sodium azide was used as the extraction solution in this study. The detection sensitivity was found to be influenced by the volume of the extract solution. A lower volume of extract solution resulted in higher sample concentration, thereby improving sensitivity. We conducted experimental evaluations to determine the optimal volume. We observed that the nasopharyngeal swab absorbed approximately 100 µL of the extract solution upon immersion. To ensure there was sufficient extract solution (around 120 µL) for the subsequent hybrid-LFA process and to account for the high viscosity of the nasopharyngeal specimen that may lead to a decrease in the volume of the extracted solution, we set the extract solution volume at 300 µL. Furthermore, the accuracy of the detection result was influenced by the sampling position within the nasal cavity. If the sampling position was too shallow, there was a risk of obtaining a false negative result due to insufficient protein concentration. To address this, each nasopharyngeal swab provided to volunteers was marked with the corresponding sampling depth, ensuring accurate positioning during sample collection. The hybrid ELAAA was employed for quantitative analysis of nasopharyngeal samples obtained from volunteers, spiked with concentrations ranging from 0.5 to 1000 ng/mL. It is reported that the N protein concentrations at 1000 ng/mL and 0.5 ng/mL approximately correspond to cycle threshold (Ct) values 15 and 33, respectively [41]. Additionally, the Ct values ranging from 15 to 33 clinically represent different infection levels from highly contagious to mildly infected individuals [41]. This clinically relevant range allows us to evaluate the assay's performance across a variety of viral loads encountered in real-world clinical patients, enhancing the applicability and significance of our findings for sensitive and accurate SARS-CoV-2 detection in nasopharyngeal samples. The obtained results demonstrated a high recovery rate along with a low CV value, as shown in Table 1. In addition, the hybrid-LFSs were utilized for qualitative analysis of samples from infected volunteers. The performance of the hybrid-LFS was validated using commercial antigen rapid test strips, and the results are presented in Additional file 1: Table S4. Remarkably, even with a limited number of positive samples, the hybrid-LFSs exhibited 100% specificity and sensitivity, affirming their exceptional reliability and accuracy. The above findings clearly demonstrate the practical applicability of hybrid ELAAA and hybrid-LFS for the detection of nasopharyngeal samples. This study offers a valuable rapid on-site detection method that regulatory agencies can utilize to effectively monitor and prevent disease hazards.

## Conclusions

In this study, we successfully expressed and utilized the SARS-CoV-2 recombinant nucleocapsid protein to generate aptamers and monoclonal antibodies. Subsequently, we established the enzyme-linked aptamer-antibody sandwich assay and hybrid lateral flow strip for the detection of SARS-CoV-2 antigen in nasopharyngeal samples. The hybrid ELAAA exhibited a remarkable detection limit of 0.1 ng/mL and demonstrated excellent stability for over one year when stored at 4 °C. Similarly, the hybrid-LFS achieved a visual detection limit of 0.1 – 0.5 ng/mL, with a test line intensity of 5 RLU in just 10 min, and maintained stability for over one year when vacuum-packaged. Furthermore, the analysis of spiked nasopharyngeal samples using the hybrid ELAAA yielded a high recovery rate, indicating its reliable performance. Moreover, the hybrid-LFS displayed high sensitivity and specificity when analyzing nasopharyngeal samples from infected and healthy volunteers. Therefore, both of these novel methods have the potential to significantly contribute to the prevention of the spread of SARS-CoV-2 and control of infectious diseases.

### Supplementary Information

**Additional file 1: Table S1.** Comparison of commercial antibodies specific SARS-CoV-2 N protein using iELISA. **Table S2.** Comparison of commercial ELISA kit for SARS-CoV-2 antigen detection. **Table S3.** Comparison of commercial rapid test strip for SARS-CoV-2 antigen detection. **Table S4.** Performance indicators of hybrid lateral flow strip for infected and healthy volunteers. **Fig. S1.** Western blotting analysis of expression SARS-CoV-2 N protein using commercial anti-SARS-CoV-2 N protein antibodies. **Fig. S2.** The stability analysis of (A) the hybrid ELAAA was conducted throughout its storage period under 4 °C. The results represent the mean ± SD of absorbance value obtained from three independent replicates. (B-C) the hybrid-LFS was conducted throughout its storage period under vacuum packaging. The results represent the mean ± SD of test line intensity (RLU) obtained from three independent replicates. **Fig. S3.** Optimization of the Hybrid-LFA. (A) Comparison of the pH value of conjugation buffer. Hybrid-LFA conditions include 20 nm gold nanoparticles with 150 µg of mAb in this evaluation. (B) Comparison of the particle size of the gold nanoparticle. This assessment involves conjugation buffer (pH 5.5) and 150 µg of mAb under hybrid-LFA conditions. (C) Comparison of the quantity of antibodies on the surfaces of gold nanoparticles. The examination is conducted using conjugation buffer (pH 5.5) and 40 nm gold nanoparticles within hybrid-LFA settings. All compared groups utilize samples containing 100 ng/mL SARS-CoV-2 N protein and 0 ng/mL SARS-CoV-2 N protein.

## Data Availability

All data used to generate these results are available in the main text and supplementary data.
