# Supplementary material for: Novel enzyme-linked aptamer-antibody sandwich assay and hybrid lateral flow strip for SARS-CoV-2 detection
Source: J Nanobiotechnology. 2024 Jan 3;22:5. doi: 10.1186/s12951-023-02191-9 (PMC10762915; doi:10.1186/s12951-023-02191-9)
Supplement: Supplementary file 1 — Additional file 1: Table S1. Comparison of commercial antibodies specific SARS-CoV-2 N protein using iELISA. Table S2. Comparison of commercial ELISA kit for SARS-CoV-2 antigen detection. Table S3. Comparison of commercial rapid test strip for SARS-CoV-2 antigen detection. Table S4. Performance indicators of hybrid lateral flow strip for infected and healthy volunteers. Fig. S1. Western blotting analysis of expression SARS-CoV-2 N protein using commercial anti-SARS-CoV-2 N protein antibodies. Fig. S2. The stability analysis of (A) the hybrid ELAAA was conducted throughout its storage period under 4 °C. The results represent the mean ± SD of absorbance value obtained from three independent replicates. (B-C) the hybrid-LFS was conducted throughout its storage period under vacuum packaging. The results represent the mean ± SD of test line intensity (RLU) obtained from three independent replicates. Fig. S3. Optimization of the Hybrid-LFA. (A) Comparison of the pH value of conjugation buffer. Hybrid-LFA conditions include 20 nm gold nanoparticles with 150 µg of mAb in this evaluation. (B) Comparison of the particle size of the gold nanoparticle. This assessment involves conjugation buffer (pH 5.5) and 150 µg of mAb under hybrid-LFA conditions. (C) Comparison of the quantity of antibodies on the surfaces of gold nanoparticles. The examination is conducted using conjugation buffer (pH 5.5) and 40 nm gold nanoparticles within hybrid-LFA settings. All compared groups utilize samples containing 100 ng/mL SARS-CoV-2 N protein and 0 ng/mL SARS-CoV-2 N protein. [file 12951_2023_2191_MOESM1_ESM.docx]

Novel Enzyme-linked Aptamer-Antibody Sandwich Assay and Hybrid Lateral Flow Strip for SARS-CoV-2 Detection

Shih-Wei Wu^1^, Ying-Ju Chen^2^, Yu-Wen Chang^3^, Cheng-Yang Huang^3^, Biing-Hui Liu^1^*, Feng-Yih Yu^3,4^*

**Materials**

Polyvinylidene fluoride (PVDF) membrane was purchased from Pall Corporation (New York, USA). Glutaraldehyde, 1-Ethyl-3-[3-dimethylami- nopropyl]-carbodiimide hydrochloride (EDC), N-hydroxysuccinimide (NHS), isopropyl-β-thiogalactopyranoside (IPTG), Ammonium persulfate (APS), TEMED, trisodium citrate, cell dissociation sieve-tissue grinder kit, sodium azide, proclin, 2-mercaptoethanol, bromophenol blue, glycerol, imidazole, potassium chloride, brilliant Blue R, phenylmethylsulfonyl fluoride (PMSF), sodium chloride, sodium dodecyl sulfate (SDS), gold (III) chloride trihydrate, sodium tetrahydridoborate, sodium bicarbonate, HAT media supplement and HT media supplement were purchased from Sigma (St. Louis, USA). Sodium phosphate dibasic, sodium phosphate monobasic, glycine, boric acid and ammonium sulfate were obtained from J.T Baker (Phillipsburg, U.S.A). Methanol, tween 20, sodium carbonate, borax, magnesium chloride, hydrogen chloride, ethanol and immobilon western chemiluminescent HRP substrate were purchased from Merck (Darmstadt, Germany). mPEG-carboxymethyl was obtained from Laysan Biotech Inc. (Arab, Alabama). HRP substrate solution 3,3’,5,5’-tetramethylbenzidine (TMB) were purchased from Neogen Corp (Lexington, KY). Complete Freund's adjuvant, Incomplete Freund's adjuvant, CarboxyLink™ coupling gel, anti-mouse-HRP, streptavidin-HRP, DEPC (Diethyl pyrocarbonate) water, DMEM (Dulbecco's modified eagle medium) and Gibco^®^ Qualified FBS (Fetal bovine serum) were obtained from Thermo scientific (East Grinstead, UK). Nickel-NTA His-bind Agarose resin was purchased from General Electric Company (Boston, U.S.A). PowerPol 2X PCR mix was purchased from Abclonal Technology (Massachusetts, U.S.A).

**Indirect ELISA (iELISA) procedure**

To screen hybridomas secreting specific antibodies against SARS-CoV-2 N protein, the solid-phase of the microplate was coated with 100 µL of N protein (1 µg/mL) and incubated at 37°C for 1 hour. After the incubation, the plate was washed four times with PBST (350 µL per well). Subsequently, 200 µL of BSA-PBS (200 µL per well) was added and incubated at 37°C. After 30 minutes, the plate was rewashed. Next, 100 µL of hybridoma culture medium (per well) was added and incubated at 37°C for 1 hour. Following this incubation, the plate was washed four times with PBST. Next, the anti-mouse IgG-HRP conjugate (diluted 1:5000 with BSA-PBST) was added to each well and incubated at 37°C for 1 hour. The plate was then washed with PBST. Finally, 100 µL of TMB substrate solution was added, and the color development was allowed to proceed for 30 minutes. The reaction was terminated by adding 100 µL of 1 N HCl to each well. The absorbance value at 450 – 650 nm was measured using an ELISA reader.

**Amplification variable-region genes of monoclonal antibody**

The total RNA of mAb-6F6E11 was extracted from hybridoma cells using the TOOLSmart RNA Extractor (Toolsbiotech, Taiwan). Subsequently, cDNA synthesis was performed through reverse transcription using the SuperScript™ IV Reverse Transcriptase (Invitrogen, USA) and Oligo(dT) 18 reverse primer (Mission Biotech, Taiwan). The amplification of the V_H_ chain employed the RS V_H_ forward primer mixture and Lin V_H_ reverse primer mixture, whereas the amplification of the V_L_ chain utilized the Lin V_L_ forward primer mixture and RS V_L_ reverse primer mixture, as described in a previous report [1]. PCR amplification was carried out with an initial denaturation step at 94°C for 5 minutes, followed by 35 cycles of denaturation (94°C, 30 seconds), annealing (50°C, 30 seconds), and extension (72°C, 30 seconds), with a final extension at 72°C for 10 minutes. The PCR products were resolved through 1.5% agarose gel electrophoresis, and the DNA was subsequently eluted using the DNA Extraction Kit (Yeastern Biotech, Taiwan).

The purified V_H_ and V_L_ gene fragments, which contained linker overhangs, were then joined together using equimolar concentrations of V_H_ and V_L_ gene fragments via overlap extension PCR. For the construction of the single-chain variable fragment (ScFv), the RS V_H_ forward mixture and RS V_L_ reverse mixture were utilized. The PCR conditions were the same as previously described, except for the annealing temperature, which was set at 63°C for 1 minute. The assembled ScFv gene was resolved on a 1.5% agarose gel and eluted using the DNA Extraction Kit. The purified ScFv DNA was cloned into a TA vector using the T&A^TM^ cloning kit and transformed into *E. coli* DH5α. Plasmid DNA was isolated from individual clones using the HiYield plasmid mini kit, and the DNA sequences were determined by Mission Biotech.

**Table S1.** Comparison of commercial antibodies specific SARS-CoV-2 N protein using iELISA

| **Vendor Name (Product No.)** | **Titration^a^** |
| --- | --- |
| This study (3A5D12) | 1:10000 |
| This study (6F6E11) | 1:25000 |
| Commercial Antibody #1 | 1:4500 |
| Commercial Antibody #2 | 1:8000 |

^a.^The dilution factor of N protein at a chromatic time of 15 min and an absorbance value of 1.5, with antibody concentration set at 0.5 µg/mL.

**Table S2.** Comparison of commercial ELISA kit for SARS-CoV-2 antigen detection

| **Vendor Name** | **Detection limit (ng/mL)** | **Procedure time** |
| --- | --- | --- |
| This study | 0.1 | 40 – 50 min |
| Commercial ELIAS kit #1 | 0.78 | 4 h |
| Commercial ELIAS kit #2 | 0.07 | 4 – 5 h |
| Commercial ELIAS kit #3 | 0.1 | 4 h |

**Table S3.** Comparison of commercial rapid test strip for SARS-CoV-2 antigen detection

| **Vendor Name** | **Visual detection limit (ng/mL)^a^** |
| --- | --- |
| This study | 0.1 – 0.5 |
| Commercial Rapid test strip #1 | 0.5 – 1 |
| Commercial Rapid test strip #2 | 100 – 500 |
| Commercial Rapid test strip #3 | 10 - 25 |
| Commercial Rapid test strip #4 | 0.5 – 1 |

^a.^Each analysis proceeded using an extract solution from this study.

**Table S4.** Performance indicators of hybrid lateral flow strip for infected and healthy

volunteers

|  | | **This study** | | **Total** |
| --- | --- | --- | --- | --- |
|  |  | Positive | Negative |  |
| **Commercial**  **immunostrip** | Positive^a.^ | 5 | 0 | 5 |
|  | Negative | 0 | 10 | 10 |

^a.^Each positive sample was verified by the physician.


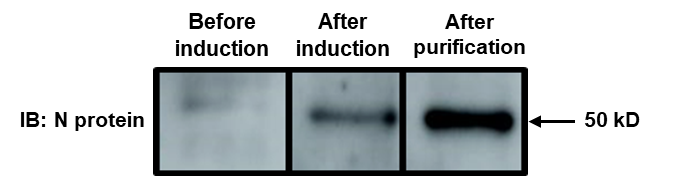


**Fig. S1** Western blotting analysis of recombinant SARS-CoV-2 N protein using the

commercial antibody specific to SARS-CoV-2 N protein (LEADGENE Biomedical

Inc., Taiwan).

| **(A)** | |
| --- | --- |
| **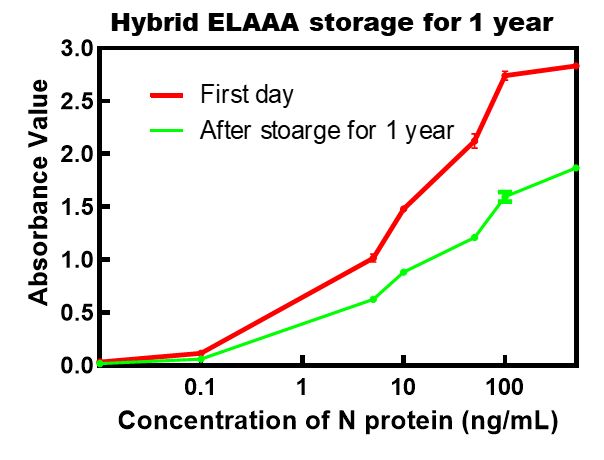** | |
| **(B)** | **(C)** |
| **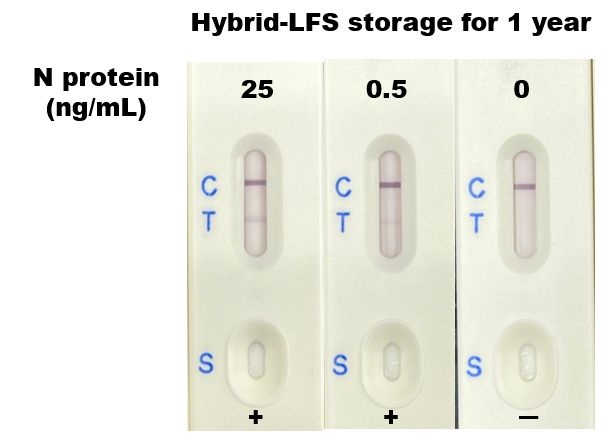** | **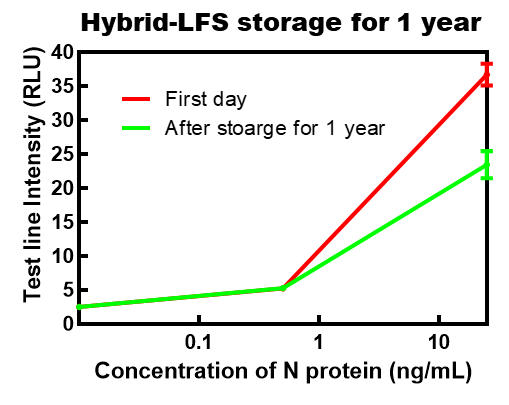** |

**Fig. S2** The stability analysis of (A) the hybrid ELAAA was conducted throughout its storage period under 4°C. The results present the average of absorbance value in each repetition analysis. (B-C) the hybrid-LFS was conducted throughout its storage period under vacuum packaging. The results present the average of test line intensity (RLU) in each repetition analysis.

| **(A)** | **(B)** |
| --- | --- |
| **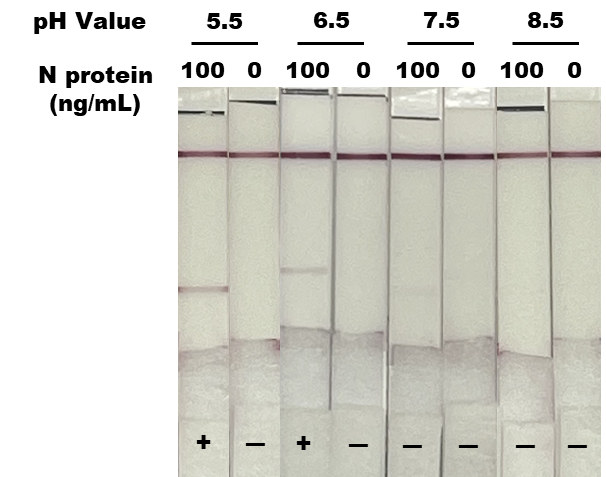** | **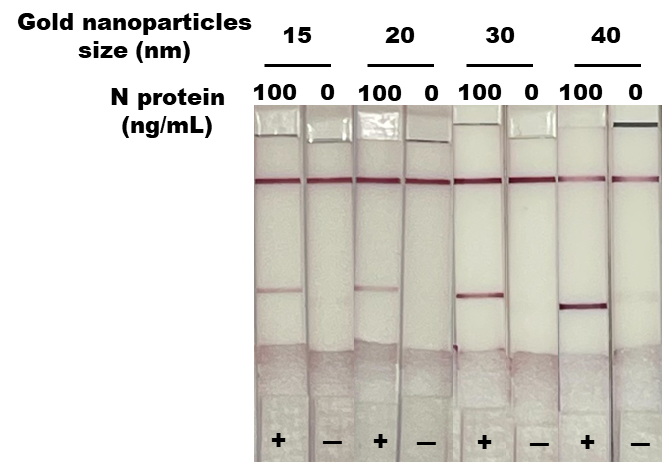** |
| **(C)** | |
| **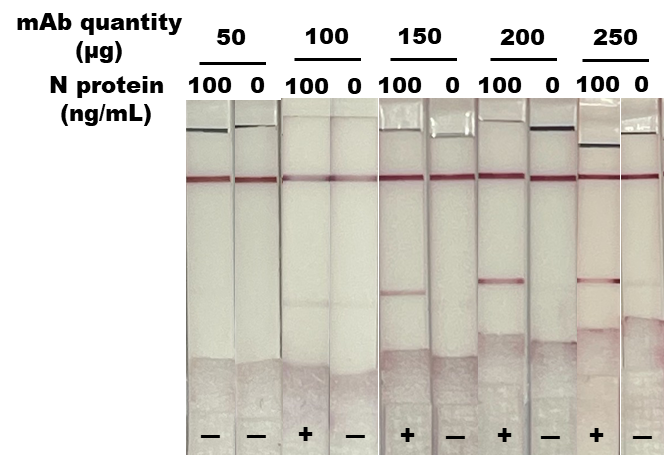** | |

**Fig. S3** Optimization of the Hybrid-LFA. (A) Comparison of the pH value of conjugation buffer. Hybrid-LFA conditions include 20 nm gold nanoparticles with 150 µg of mAb in this evaluation. (B) Comparison of the particle size of the gold nanoparticle. This assessment involves conjugation buffer (pH 5.5) and 150 µg of mAb under hybrid-LFA conditions. (C) Comparison of the quantity of antibodies on the surfaces of gold nanoparticles. The examination is conducted using conjugation buffer (pH 5.5) and 40 nm gold nanoparticles within hybrid-LFA settings. All compared groups utilize samples containing 100 ng/mL SARS-CoV-2 N protein and 0 ng/mL SARS-CoV-2 N protein.

**References**

1. Singh PK, Agrawal R, Kamboj DV, Gupta G, Boopathi M, Goel AK, Singh L. Construction of a single-chain variable-fragment antibody against the superantigen Staphylococcal enterotoxin B. Appl Environ Microbiol. 2010;76:8184-8191
